# Crossing the gates of Babylon: Brain-penetrating enzyme replacement for lysosomal disorders

**DOI:** 10.1016/j.omtm.2023.07.008

**Published:** 2023-08-10

**Authors:** Guilherme Baldo

**Affiliations:** 1 Departamento de Fisiologia, Universidade Federal do Rio Grande do Sul, Porto Alegre, Rio Grande do Sul 90035-903, Brazil; 2 Casa dos Raros, R. São Manoel, 730 Santa Cecília, Porto Alegre, RS 90610-261, Brazil

Situated in modern-day Iraq, the ancient city of Babylon was located in a fertile region and at the crossroads of important trade routes. A significant architectural wonder of Babylon was its grand entrances known as the Babylon Gates. These gates served as more than mere portals. Firstly, they functioned as points of entry into the city, controlling the flow of people and goods. Secondly, they acted as defensive fortifications, protecting the city against potential invaders. With similar functions, the “gate” that gives access to our brain is the blood-brain barrier (BBB).

The BBB is a highly specialized protective system that separates the circulating blood from the brain tissue. It is composed of a tightly packed layer of endothelial cells lining the walls of the brain’s blood vessels. It restricts the entry of harmful substances, such as toxins, pathogens, and certain medications, into the brain while allowing the passage of essential nutrients and waste products.

The presence of the BBB is one of the main challenges faced by enzyme replacement (ERT) strategies designed to treat multisystemic disorders with brain involvement, including Gaucher disease types 2 and 3 and most mucopolysaccharidoses (MPS), for example. Since these enzymes do not cross the BBB, they do not reach the neuron cells affected in such disorders. As a consequence, treated patients still exhibit progressive deterioration of the brain function.^1^

Many approaches have been tested to circumvent the barrier and deliver the enzymes to neuronal cells. For more than a decade, reports of intrathecal enzyme replacement in such disorders such as MPS type I and type II have been described and, despite improvements in biochemical parameters, including reduction in glycosaminoglycan levels in the cerebrospinal fluid, changes from baseline in functional evaluations such as the Vineland Adaptive Behavioral Scales were similar between treated and untreated patients, and therefore, data from randomized clinical trials were considered insufficient to meet the evidentiary standard to support regulatory filings for both disorders^2^^,^^3^

Here, Kida et al.^4^ report efficacy and safety of JR-171, a fusion protein comprising humanized anti-human transferrin receptor antibody Fab and IDUA, the enzyme deficient in MPS type I. The fusion protein uses the binding of the antibody to the transferrin receptor, which is abundant in the endothelial cells of the BBB, to carry the IDUA across the membrane. The enzyme then reaches the brain and is able to clear storage of glycosaminoglycans within neurons and to improve cognitive function, assessed by Morris water maze test. Furthermore, they observed no adverse effects, even at higher dose, in cynomolgus Monkeys.

Both the Food and Drug Administration and the European Medicines Agency have given JCR-171 the orphan drug designation, and clinical trials are being conducted to assess efficacy. These trials will face the difficult task of proving the efficacy of these therapies in humans. Based on the trials from intrathecal and intravenous ERT, biochemical alterations do not necessarily correlate with improved brain function. However, in the last years, studies in the animal models^5^ have shown that the heparan sulfate levels in the spinal fluid correlate with brain tissue heparan sulfate, and in theory, this should lead to improved function as well, if the enzyme is well distributed along the central nervous system and if treatments is performed early. Therefore, such biomarkers, including non-reducing end or disaccharide glycosaminoglycans, should be used in short-term clinical trials and considered for approval of these therapies, but long-term trials need to include functional parameters and brain imaging as well.

If such encouraging data from the efficacy of this ERT in preclinical studies are confirmed in clinical trials, the same platform could be used for many other similar disorders that currently have no effective treatment. Those disorders include Krabbe disease, GM1 and GM2 gangliosidoses, and metachromatic leukodystrophy, to name a few. It is certainly exciting news for this group of disorders, knowing that a merchant who is able to cross the gates that protect the brain was found. Now, it is time to find out if the goods we are taking in are actually as good as we expect them to be.
